# What Is the Proper Use of Antiseptics in Puerperal Disease?*Read before the New York State Medical Association, at the annual meeting in New York, October 10, 1888.

**Published:** 1888-11

**Authors:** Rollin Ledrue Banta

**Affiliations:** Buffalo, Fellow of the American Association of Obstetricians and Gynecologists; Obstetrician to the Buffalo Maternity Hospital; 358 South Division Street


### Buffalo Medical and Surgical Journal

Vol. XXVIII. NOVEMBER, 1888. No. 4

### Original Communications.

### *WHAT IS THE PROPER USE OF ANTISEPTICS IN PUERPERAL DISEASE?**

By ROLLIN LEDRUE BANTA, M. D., Buffalo,

Fellow of the American Association of Obstetricians and Gynecologists; Obstetrician to the Buffalo Maternity Hospital.

"What plan of antiseptic treatment can be employed with a large degree of success in each of the several forms of the disease?

"Does every rise of temperature above 100° F. in the puerperal woman constitute an indication for immediate resort to irrigation?

"When should irrigation be intra-vaginal, and when intra-uterine?

"When irrigation is employed, how often should it be done, and when discontinued?

"What hygienic, medicinal, and dietetic treatment should be used in addition to the local antiseptic measures? To what extent should alcoholic stimulants and antipyretics be used?"

About ten years ago it was my misfortune to have in my practice a series of puerperal fever cases. Notwithstanding the strictest antiseptic precautions (the value of the mercuric compounds as germicides were not known to me at that time), one case followed another—not consecutively, but skipping over sometimes two or three, only to reappear in a fourth or fifth. In desperation I had my arm done up for a pretended Colles' fracture, in order to have an excuse for giving up my obstetrical, while retaining my general, practice. After the lapse of four weeks, another effort was made to attend a confinement, with the result

* Read before the New York State Medical Association, at the annual meeting in New York, October 10, 1888.

of having two fresh cases on my hands. Of course, nothing now remained but to stop practice entirely. I accordingly did so, and resumed again at the expiration of six weeks, having thus successfully stamped out the disease.

Since that time puerperal septicemia, in its various forms, now and then appears in my practice; and occasionally there is a death. But it is no unusual thing for me to have a hundred consecutive confinements without any symptoms that would cause a day's uneasiness. Perhaps a riper experience has had something to do with this success; but, in my opinion, strict cleanliness in all things, as far as possible—and it is impossible to obtain that cleanliness without the use of a powerful antiseptic—has undoubtedly had far more to do with these results.

However, my share in this discussion is not to discourse upon how to keep away puerperal septicemia, but rather upon how to treat the disease. Although the temptation is great to add a flower to the wreath of immortality, which forever will encircle the brow of Semelweis, and give one word of praise to Tarnier, who first used the mercuric chloride; still, it is my duty to leave that task to my associates, who will give credit where it is due, and to proceed to give what, in my opinion, seems to be the best plan of treatment for puerperal septicemia.

The great object in the surgery of to-day in performing an operation is, to exclude all septic material. This is especially true in modern obstetrics. Every precaution should be taken to keep away the poison germ; or, if it has gained a foothold, to destroy it as soon as possible.

The genital tract after labor has all the conditions favorable for the growth of bacteria. The abraided mucous surfaces, saying nothing of a lacerated os, or torn perineum, afford ample opportunity for their passage into the general system. If microbes of a malignant nature have gained entrance to the parturient canal, either just before, during, or after childbirth, certain symptoms occur, which usually manifest themselves at say from thirty to seventy hours afterwards. These symptoms, to the experienced obstetrician, prove that infection has taken place.

Yet a case may occur occasionally in which there may be a doubt as to the proper steps with which to begin treatment.

When once we are sure that the poison is there, what is the best way to prevent it from doing further mischief? Certainly only one plan naturally suggests itself, and that is, to clean out the vagina and uterus, and to destroy with germicides any poisonous elements that remain. Often simply washing out the vagina with an antiseptic solution, will produce an immediate improvement in the symptoms of the patient; but if there should be no benefit after twenty-four, and, at the latest, forty-eight hours, it is better to proceed at once to attack the whole of the parturient canal.

To do this, three things are necessary: A Davidson's syringe or fountain irrigator, an antiseptic solution, and a proper tube.

Perhaps a word will not be out of place regarding the tube, which with me has the preference over all others. This is a hard-rubber tube, that can be bought at a slight cost at any rubber store, in the form of a knitting needle. With the aid of boiling water, a file, and a sharp-pointed awl or knife, it can, in a very few minutes, be made into any shape or form. On account of its cheapness it can be thrown away after using once; and, by so doing, we can always be certain of having a clean tube.

Everything ready, the patient is brought to the edge of the couch in the forceps position, with a rubber blanket underneath her, so arranged as to guide the water into a small tub, placed partly under the bed. The water should be hot enough to bear comfortably the elbow in it. After expelling the air from the tube, pass it gently to the fundus of the uterus, then withdraw it slightly, so as to be sure that the distal end is not in a uterine sinus; work the syringe with the slightest amount of force, using at least a quarter of the solution, or, better still, a gallon, watching that it flows back from the womb into the vagina, and thence into the tub.

As to the antiseptic to be used: After trying quite a number of others, I have returned to the mercuric chloride as the best. True, there have been some fatal cases reported from its use, but it has never been knowingly a source of danger to me, for the reason, perhaps, that I never use it stronger than one part to 4,000, and oftener as weak as one part to 10,000. If the injections are going to do any good, they will do so at once. There is a fall of the temperature, the pulse becomes slower, and the enlarged and softened womb becomes smaller after each injection. Indeed, there is a general improvement. This irrigation may be used at first three times each day, and then be dropped to two, and finally to one, as may be found necessary.

If there is no improvement, or only a very slight one, and the womb remains in a soft and flabby condition, and at each treatment small particles come away, it is evident that irrigation alone will not do the work, but needs more assistance. This can be obtained by the use of the curette, which with me has been productive of a great deal of good, and never of harm. Used in the same position as mentioned above, it is surprising what a large amount of débris can be scraped out of a uterine cavity after it has been thoroughly washed out for days; surprise will often give way to regret, to think that the bacterial nests had not been destroyed long before.

For quite a number of years, when attending miscarriages, it has been my custom to empty the womb by means of the curette and the small placenta forceps, always supplementing the treatment by the antiseptic douche; and if the os is patulous enough to allow the finger to take the place of the curette, providing there has been no prior infection, almost perfect impunity from septic troubles can be promised.

The reason is simple enough. One hand having been introduced into the vagina, and one or more fingers into the cervix (nothing can take the place of the educated finger), the other hand placed on the outside, pushing the uterus down sufficiently to reach the fundus, and even each horn, all parts may be exposed and scraped out, and the cavity so left that it can be put into a perfectly antiseptic condition. In that condition no baneful results can follow.

So it is in the treatment of puerperal septicemia. When infection has once taken place, the great object is to remove quickly, and as thoroughly as possible, the matters that do not belong to the parturient canal, and make innocuous what remains. If we fail, it is either because the poison has entered through some other avenue of the body, or because the main army of the destructive germs has passed into the system, and only the rear guard is left for us to fight against by local treatment.

The older obstetricians taught that the genital organs of a woman after labor were intolerant of manipulation. That is only true now when the manipulator is ignorant or unskillful.

How often does it happen that septic troubles follow the removal of the retained placenta; and why is this so? Simply because when nature is unnecessarily interfered with, or requires the aid of art, she requires in no uncertain way that her methods shall be closely followed. In other words, when nature removes the afterbirth, she does it thoroughly, and leaves no poison germs behind; if artificial aid is required, and does not do it as thoroughly as she, a heavy penalty must be paid.

Without doubt, many cases of puerperal septicemia will get better without the aid of antiseptic treatment; but it is my firm conviction, where one will pull through, depending only upon the older methods, ten will die. It is understood, of course, that the severer types of the disease is meant.

Many times after labor, without any appreciable cause, there is a high rise of temperature, which soon passes away without any treatment, or upon the administration of an antiseptic. It is only the temperature that lasts longer than twenty-four hours that is a source of anxiety. High temperature is not always a forerunner of evil. Other symptoms are to be taken into consideration, and it would be injudicious, to say the least, to resort to irrigation without waiting for further developments. But, in my experience, early irrigation has never been attended with any evil results; on the contrary, in quite a number of cases, delay has been a matter of regret.

If, as has been said before, the vaginal douche does no good, after having been used at intervals of four hours, for twenty-four, or, at the furthest, forty-eight hours, the intra-uterine douche should be substituted at once, and used at least three times in the twenty-four hours, and continued so long as good results are obtained, until the patient is cured.

If the trouble proceeds from the vagina, and is there alone, intra-vaginal injections will soon change the bad symptoms into good ones; if, however, the mischief is in the uterus, intra-uterine irrigation may have to be aided by the curette. If, after that procedure, followed by injections for two or three days, there is no benefit, you may be sure it is of no use to continue them. The poison has entered into the general system, and is beyond the control of local measures, or infection has come through some other channel of the body, and has to be treated as such.

If the intra-uterine injections do good, the pulse will very soon become slower and the temperature lower; the womb will contract and gradually become smaller; the lochia will become inoffensive to the nostril, and the injections may be discontinued just as soon as there is a certainty that there is no dangerous material left in the genital tract.

Regarding the general treatment of the disease under consideration, although much could be said, it is my intention to say very little.

One of the first things to do is to get the patient into a large room with plenty of fresh air, but no draughts.

Give a light but nutritious diet.

The obstinate vomiting may usually be brought under control by cocaine, or, better still (if it alone will do it), by peptonised milk.

Antipyretics should be given often enough and in sufficient quantities to keep the temperature below 100° F. Opium (which, in some of the inflammatory types of the disease, is the sheet-anchor of hope) should be given in doses large enough to quiet pain and produce sleep, though, perhaps, chloral would be the better remedy to produce the latter result.

Early in the disease the antipyretics not only lower the temperature, but produce copious diaphoresis, which is, without question, a valuable help to nature's method of throwing off the disease, after it has gained entrance into the general system. Later they seem to weaken the heart's action, and then it is wise to give alcohol in some of its various forms, which, "when given, fully saturates the system, sustains the strength of the patient, and by its affinity for fat, penetrates the cells, checks metamorphosis of tissue, and retards bacterial activity."

358 SOUTH DIVISION STREET.

